# Non-contact physiological monitoring of post-operative patients in the intensive care unit

**DOI:** 10.1038/s41746-021-00543-z

**Published:** 2022-01-13

**Authors:** João Jorge, Mauricio Villarroel, Hamish Tomlinson, Oliver Gibson, Julie L. Darbyshire, Jody Ede, Mirae Harford, John Duncan Young, Lionel Tarassenko, Peter Watkinson

**Affiliations:** 1 Institute of Biomedical Engineering, Department of Engineering Science, University of Oxford, Oxford, UK; 2 NIHR Biomedical Research Centre, Oxford, UK; 3 Oxehealth Ltd., The Oxford Science Park, Magdalen Centre North, Oxford, UK; 4 Kadoorie Centre for Critical Care Research and Education, Nuffield Department of Clinical Neurosciences, University of Oxford, Oxford, UK; 5 Oxford University Hospitals NHS Trust, Oxford, UK

**Keywords:** Translational research, Biomedical engineering

## Abstract

Prolonged non-contact camera-based monitoring in critically ill patients presents unique challenges, but may facilitate safe recovery. A study was designed to evaluate the feasibility of introducing a non-contact video camera monitoring system into an acute clinical setting. We assessed the accuracy and robustness of the video camera-derived estimates of the vital signs against the electronically-recorded reference values in both day and night environments. We demonstrated non-contact monitoring of heart rate and respiratory rate for extended periods of time in 15 post-operative patients. Across day and night, heart rate was estimated for up to 53.2% (103.0 h) of the total valid camera data with a mean absolute error (MAE) of 2.5 beats/min in comparison to two reference sensors. We obtained respiratory rate estimates for 63.1% (119.8 h) of the total valid camera data with a MAE of 2.4 breaths/min against the reference value computed from the chest impedance pneumogram. Non-contact estimates detected relevant changes in the vital-sign values between routine clinical observations. Pivotal respiratory events in a post-operative patient could be identified from the analysis of video-derived respiratory information. Continuous vital-sign monitoring supported by non-contact video camera estimates could be used to track early signs of physiological deterioration during post-operative care.

## Introduction

Clinical complications following surgery remain a major concern worldwide^1^, therefore admission to the intensive care unit (ICU) following major surgery is standard practice in many healthcare systems. Patients discharged from an ICU to the ward following a post-operative ICU admission are at high risk of adverse events^2^.

In-hospital adverse events may be prevented with earlier detection of physiological deterioration followed by prompt intervention^3–5^. Early detection of deterioration in post-ICU ward patients has been shown to reduce the rate of ICU readmission^6^, which is associated with worse outcomes^7^. The incidence of adverse events, such as cardiac arrest or unexpected admission to the ICU, could also be reduced^3,8^, which in turn is associated with improved outcomes^9,10^.

Patient care within hospital settings relies on a system of escalation based on a weighted assessment of patients’ vital signs. The monitoring of vital signs, including heart rate (HR), respiratory rate (RR), peripheral blood oxygen saturation (SpO_2_), blood pressure and core temperature provides valuable insights into the overall condition of the patient^11,12^. Patterns of critical illness are often preceded by periods of physiological abnormality^13,14^, detected in the recorded vital signs well before patients show physical signs of distress^15,16^. Effective patient monitoring strategies, therefore, aim to detect abnormal patterns in the vital signs as often and as soon as they occur so that acute care for deteriorating patients can be escalated^2,17^.

Clinical staff evaluates vital signs with a measurement frequency ranging from hourly to 12 h, depending on the acuity level of the ward, and individual patient condition^18,19^. High-acuity patients such as those in the ICU are monitored continuously using automated bedside monitors so that acute changes can be detected and acted upon in a prompt manner. Heart rate and cardiac function are monitored through electrocardiography (ECG). A pulse oximeter attached to a well-perfused part of the body, such as a finger or an earlobe, is often used to provide non-invasive estimates of SpO_2_ via two-wavelength photoplethysmography (PPG). Respiratory rate is measured using Impedance Pneumography (IP). IP is a convenient method if patients are already monitored by ECG as it relies on the existing set of electrodes. High-acuity patients may also require invasive monitoring, including arterial cannulation to continuously monitor the blood pressure.

Post-operative ward patients are typically less acute. This is reflected in the level of monitoring they receive, with most patients’ vital signs measured manually every 1–4 h for the immediate post-operative period. Ward level measurements of vital signs are usually performed using a portable monitor, such as DINAMAP^®^, which incorporates a pulse oximeter and a blood pressure cuff^20^. Respiratory rate is counted manually. The frequency of manual observations can be increased to multiple measurements per hour for post-operative patients who require intensive monitoring. Continuous monitoring equipment may also be used. Conventional contact monitors are, however, poorly tolerated by patients^21^. They limit patient mobility, rehabilitation and are uncomfortable to wear over prolonged periods of time^22^. One previous study that aimed to monitor patients in acute medical and surgical wards for 72 h found that a significant proportion of patients declined to be monitored for the entirety of this period^21^. Numerous monitoring wires are cumbersome and make patient transfers more difficult^23^. Nursing staff have reported many staff-hours spent troubleshooting current monitoring systems, with common causes of monitoring failures including sensor disconnection, poor vital-sign readings, and equipment malfunctio^24^. Poor probe contact has been identified as the leading cause of false respiratory alarms in patients monitors^25^.

For continuous vital-sign monitoring to be feasible in hospitalised patients it must maintain measurement accuracy while being widely acceptable to patients and staff. A way of achieving this is by using less intrusive monitors instead of the conventional contact-based sensors^26^. There is growing support that video camera-based vital-sign monitoring may fulfil this role^27–30^, a view reinforced by recent findings that such monitoring is feasible in several hospital environments^31–35^.

Photoplethysmographic imaging (PPGi) is based on the principle that a cardiorespiratory waveform may be extracted from the ambient light reflected from an area of exposed skin using conventional camera devices^36,37^. Numerous studies in controlled laboratory environments have demonstrated that heart rate and respiratory rate can be derived from the PPGi waveform with a high degree of accuracy^38,39^. Work published in recent years has shown that changes in SpO_2_ may also be derived^40–42^. Methods continue to be developed which improve the quality of the PPGi waveform extracted from video feeds^43,44^. Recent efforts to improve the robustness of non-contact monitoring to motion artefacts have also been reported^45–47^.

While there are clear advantages to conducting experiments in controlled environments, this practice cannot emulate the challenging conditions of an ICU. There are substantial challenges that PPGi must overcome in this setting, including unforeseeable signal artefacts due to patient movement, clinical interventions or changes in the illumination from ambient light sources^31^. PPGi-based monitoring of heart rate and respiratory rate has so far been demonstrated in non-critical in-hospital areas such as the outpatient clinic^31,32^, the neonatal high dependency unit^33^, and the intra-operative environment^34,35^. While one pilot study collected early data in a post-operative ICU following elective cardiac surgery for 28.6 ± 2.8 min (per patient)^48^, the feasibility of non-contact monitoring for extended periods of time remains untested.

We designed a clinical study to assess the feasibility of using a video camera-based system to estimate heart rate and respiratory rate over clinically relevant time periods from post-surgical patients with a planned post-operative admission to the ICU in the Churchill Hospital in Oxford, UK. We aimed to establish the proportion of time that camera signals of sufficient quality for reliable vital-sign estimation could be obtained in this patient group, as well as the accuracy of the vital-sign estimates obtained from the video camera system in comparison to wired monitoring collected concurrently as per usual patient clinical care in the ICU.

## Results

Prior to their operation, 25 patients agreed to take part over the course of one year. Recording data was available for a cohort of 15 patients as 10 patients were not admitted to the ICU for one of the following reasons: patient admitted to an alternative Recovery Unit (4 patients), surgery cancelled or rescheduled (3 patients), or unavailable research staff or equipment (3 patients).

A summary of demographic data for the study cohort is included in Table 1. The average age of monitored patients was 62.2 years. Nine patients were male (60%) and six patients were female (40%). Ten patients were admitted to the ICU following maxillofacial surgery (60%), four (26.6%) following gastrointestinal (GI) tract surgery and one patient was admitted following gynae-oncological surgery. The average APACHE II score was 15.7 ± 4.5 at the time of admission to the ICU. The majority (*n* = 9) commenced study recording with a Richmond Agitation-Sedation Scale RASS < 0. All but one patient became alert (RASS = 0) within the recording period. Eight study patients were receiving airway pressure support from a bilevel ventilation device at the start of data recording. The patients were predominantly of white skin colour, with 93.3% of patients classified as having either skin type I or II on the Fitzpatrick scale^49^. Only one patient was classified as skin type III.

**Table 1:** Summary of the study population.

Total number of patients	15
Total recording time (h)^a^	233.5
Gender^b^	
Male	9 (60.0%)
Female	6 (40.0%)
Age (years)^c^	62.2 (12.4)
Clinical specialty^b^	
Maxillofacial	10 (60.7%)
Lower GI	2 (13.3%)
Upper GI	2 (13.3%)
Gynaecological/Oncology	1 (6.7%)
APACHE II score^c^	15.7 (4.5)
RASS score < 0^b^	9 (60.0%)
Ventilation status^b,d^	
Bilevel	7 (47.7%)
None	8 (53.3%)
Propofol infusion^b,d^	6 (40.0%)
Fitzpatrick skin type^b^	
I	2 (13.3%)
II	12 (80.0%)
III	1 (6.7%)
Recording time per patient (h)^a,c^	15.6 (8.7)
Day	6.8 (4.5)
Night	8.8 (5.5)

^a^ Day (08:00 to 19:59), night (20:00 to 07:59)

^b^ N (percentage from total number of patients)

^c^ mean (std)

^d^ At the start of data recording

Patients were recorded for a total of 233.5 h, with the average session lasting approximately 15.6 h. Recording time was defined as periods of concurrent recording of video and monitor data (including both heart rate and respiratory rate values). Most post-operative patients were admitted to the ICU in the evening. Each patient was monitored for an average of 6.8 day-time hours and 8.8 night-time hours, although these figures varied considerably between patients. None of the patients withdrew from the study.

### Statistical analysis

**Fig. 1:**
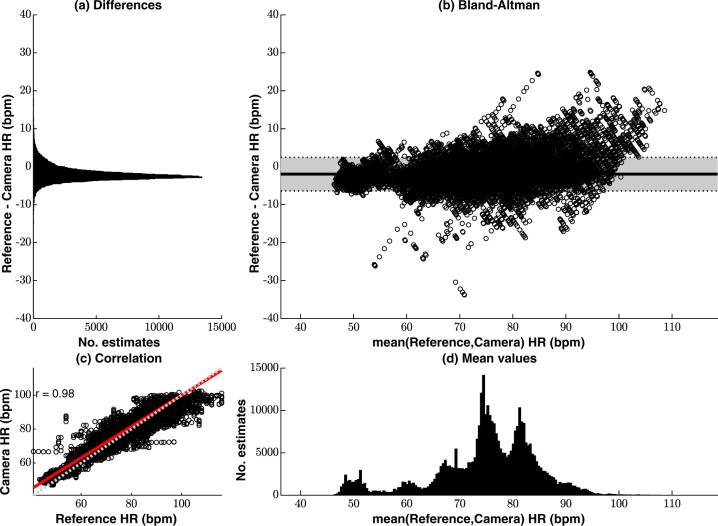
Agreement between the reference heart rate values (computed from the ECG and PPG) and the camera estimates for the valid video camera data, comprising a total estimated time of approximately 103.0 h. **a** The differences between the camera and reference monitors are normally distributed. **b** The Bland-Altman plot presents a minimal sensor bias. **c** The scatter plot shows high correlation between the two devices, with a Pearson correlation coefficient of 0.98. **d** The distribution of the mean values shows that most of the heart rate estimates are within the expected physiological range for adults.

Figure 1 shows the agreement between the reference heart rate values (computed from the ECG and PPG) and the video camera estimates for the valid video camera data from the 15 patients. The Bland-Altman plot, shown in Fig. 1b, presents minimal sensor bias (−2.0 beats/min) with small differences (95% limits of agreement at −6.4 beats/min and 2.5 beats/min) normally distributed (Fig. 1a). The distribution of the mean values in Fig. 1d, shows that heart rate values were distributed over the expected range for adults. Figure 1c shows the regression line between the two methods. The values estimated by the two devices have a Pearson correlation *r* coefficient of 0.98. (Fig. 1d).

**Fig. 2:**
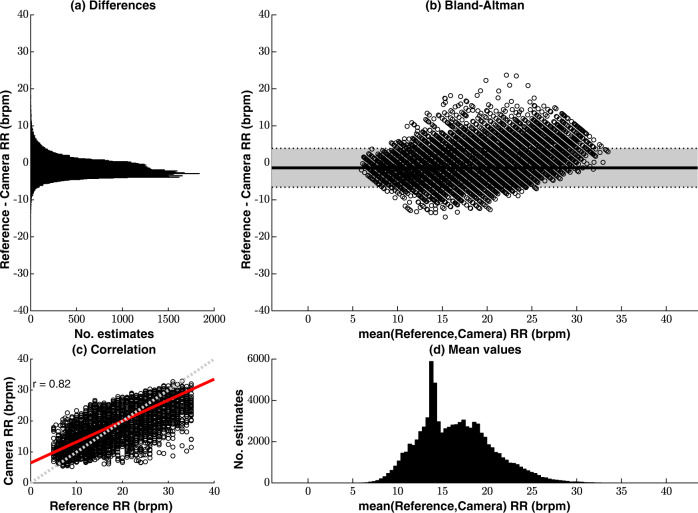
Agreement between the reference respiratory rate values (computed from the chest impedance pneumogram) and the camera estimates for the valid video camera data, comprising a total estimated time of approximately 119.8 h. **a** The differences between the two estimates. **b** The Bland-Altman plot presents negligible sensor bias. **c** The plot shows a positive correlation between the two devices, with a Pearson correlation coefficient of 0.82. **d** The distribution of the mean values shows that most of the respiratory rate estimates are within the expected physiological range for adults. brpm = breaths/min.

Figure 2 shows the agreement between the reference respiratory rate values from the chest impedance pneumogram and the video camera estimates from the valid video camera data of the 15 patients. The Bland-Altman plot, shown in Fig. 2b, presents negligible sensor bias (−1.4 breaths/min) with narrow differences across the measured range (95% limits of agreement at −6.6 breaths/min and 3.9 breaths/min), which indicate a good agreement between the two methods (Fig. 2a). The mean values of respiratory rate shown in Fig. 2d are distributed over the normal physiological range. A peak in the distribution in Fig. 2d at approximately 14 breaths/min was recorded due to the inclusion of a patient undergoing mechanical ventilation at this rate. Further details on this monitoring case are provided in our discussion of Fig. 4. Figure 2c shows the regression line between the two measurements. The values estimated by the two devices have a positive correlation coefficient *r* of 0.82.

### Cohort analysis

Table 2 presents the results of video camera-based estimation of both vital signs for each of the recorded sessions.

**Table 2:** Summary of vital-sign estimation results for all recording sessions.

Patient	Total recording time (h)	Private time (h)	Heart rate			Respiratory rate		
			Valid time	Estimated time	MAE^a^	Valid time	Estimated time	MAE^a^
			(hours, %)^b^	(hours, %)^c^	(beats/min)	(hours, %)^b^	(hours, %)^c^	(breaths/min)
1	15.2 h	2.2 h	12.4 h, 81.9%	9.0 h, 58.9%	2.1	9.2 h, 60.5%	5.5 h, 36.0%	2.1
2	27.1 h	4.9 h	20.0 h, 73.7%	9.9 h, 36.4%	2.5	21.1 h, 77.7%	11.2 h, 41.3%	1.9
3	5.8 h	2.1 h	3.8 h, 64.7%	2.5 h, 42.3%	5.2	1.7 h, 29.8%	1.3 h, 21.9%	3.1
4	18.1 h	3.4 h	14.7 h, 81.1%	6.4 h, 35.5%	2.6	16.4 h, 90.8%	12.8 h, 70.5%	2.3
5	1.3 h	0.2 h	0.9 h, 65.7%	0.6 h, 41.9%	3.0	1.1 h, 85.8%	0.8 h, 58.5%	1.5
6	15.9 h	0.6 h	15.3 h, 96.1%	3.9 h, 24.6%	2.9	11.3 h, 71.4%	9.1 h, 57.2%	2.6
7	5.5 h	0.0 h	4.3 h, 77.8%	3.5 h, 63.9%	2.5	5.6 h, 102.7%	4.8 h, 86.7%	2.7
8	16.0 h	2.6 h	13.3 h, 83.5%	9.0 h, 56.2%	2.7	13.5 h, 84.3%	11.3 h, 70.4%	1.2
9	21.1 h	0.7 h	20.3 h, 96.4%	8.7 h, 41.4%	2.0	15.4 h, 73.2%	8.8 h, 41.8%	2.7
10	26.8 h	4.4 h	22.4 h, 83.5%	14.5 h, 54.1%	2.5	21.3 h, 79.5%	13.3 h, 49.7%	2.6
11	30.4 h	4.5 h	25.9 h, 85.2%	15.8 h, 52.0%	2.5	23.6 h, 77.7%	13.0 h, 42.8%	2.7
12	19.3 h	2.9 h	13.3 h, 68.8%	8.4 h, 43.2%	2.5	17.2 h, 89.1%	9.7 h, 50.1%	2.4
13	10.1 h	2.0 h	8.1 h, 80.5%	2.4 h, 24.3%	2.5	7.4 h, 73.8%	4.9 h, 48.5%	3.0
14	14.8 h	0.3 h	14.4 h, 97.7%	4.8 h, 32.8%	2.6	13.9 h, 93.9%	6.8 h, 46.0%	3.6
15	6.2 h	0.4 h	4.7 h, 75.5%	3.6 h, 58.8%	2.7	6.9 h, 112.0%	4.9 h, 79.7%	2.5
Overall	233.5 h	31.2 h	193.7 h, 82.9%	103.0 h, 53.2%	2.5	189.7 h, 81.3%	119.8 h, 63.1%	2.4

^a^ Mean absolute error

^b^ Percentage with respect to total recording time

^c^ Percentage with respect to valid time

Periods when an opaque privacy blind was used to obscure the view of the patient during dignity-compromising situations (e.g. routine hygiene) are reported in Table 2 under private time. The percentage of private time per participant ranged from 1.4% (0.2 h) to 41.8% (2.7 h) of the total session duration.

Artefacts are a common cause of false alarms, signal loss, and inaccurate measurements in physiological monitors^50^. To establish gold-standard references against which the performance of camera-derived vital-sign estimates could be assessed, we assessed data from standard patient monitoring equipment. We used automated methods to identify and discard those monitor values found to be of poor quality according to accepted clinical standards^51^ (see Supplemental material 1). Common causes for poor-quality monitor readings included the occurrence of clinical interventions, family visits or instances when the patient was awake and active, causing motion artefacts to corrupt the contact-sensor readings. The remaining time, reported as valid time in Table 2, and comprising a total length of 193.7 h of heart rate monitoring (82.9%) and 189.7 h (81.3%) of respiratory monitoring, was considered for performance analysis.

Estimated time refers to periods of good-quality video data (as assessed using independent signal analysis). It corresponds to effective cardiorespiratory monitoring time, and is reported both in absolute time, in hours, and as proportion of the valid time. Including both day and night, heart rate estimates could be obtained for 53.2% (103.0 h) of the total valid time (193.7 h) with a mean absolute error (MAE) of 2.5 beats/min. Respiratory rate was estimated with a MAE of 2.4 breaths/min for up to 63.1% (119.8 h) of the total valid time (189.7 h).

Environments with variable illumination (such as the ICU) are susceptible to low levels of ambient light, shadows and exposure to unknown light sources. These environments pose an intrinsic challenge to PPGi technology, potentially limiting the practical implementation of video camera-based monitors. Of particular concern in the ICU environment are the periods of time during the evening and night, when fluorescent lights are often dimmed. Table 3 summarises the performance of video camera-based estimation of vital signs for two periods: day-time (08:00 to 19:59) and night-time (20:00 to 07:59).

**Table 3:** Summary of vital-sign estimation results for the day-time and night-time periods.

Period	Total recording time (h)	Private time (h)	Heart rate				Respiratory rate			
			Valid time	Estimated time	MAE^a^	*r*^b^	Valid time	Estimated time	MAE^a^	*r*^b^
			(hours, %)^c^	(hours, %)^d^	(bpm)		(hours, %)^c^	(hours, %)^d^	(brpm^e^)	
Day-time	102.0 h	16.2 h	78.5 h, 77.0%	42.6 h, 41.8%	2.7	0.96	77.0 h, 75.5%	45.1 h, 44.2%	2.6	0.81
Night-time	131.5 h	15.0 h	115.2 h, 87.6%	60.4 h, 45.9%	2.5	0.98	112.8 h, 85.8%	74.7 h, 56.8%	2.3	0.82
Overall	233.5 h	31.2 h	193.7 h, 82.9%	103.0 h, 53.2%	2.5	0.98	189.7 h, 81.3%	119.8 h, 63.1%	2.4	0.82

^a^ Mean absolute error

^b^ Pearson *r*

^c^ Measurements were classed into day-time (7:00 am to 6:59 pm) and night-time (7:00 pm to 6:59 am). Percentage with respect to total recording time

^d^ Percentage with respect to valid camera time

^e^ breaths per minute

Heart rate estimates could be obtained for 41.8% (42.6 h) of the valid day-time period (78.5 h) with a MAE of 2.7 beats/min versus 2.5 beats/min obtained for 45.9% (60.4 h) of the valid night-time period (115.2 h). Respiratory rate estimates could be obtained for 44.2% (45.1 h) of the valid day-time period (77.0 h) with a MAE of 2.6 breaths/min while a MAE of 2.3 breaths/min was obtained for 56.8% (74.7 h) of the valid night-time period (112.8 h). No meaningful difference was reported in the duration of private time between the two periods (16.2 versus 15.0 h). Bland–Altman plots for the two time periods are provided in the supplemental material.

### Noteworthy monitoring scenarios

This section presents three monitoring cases identified from the retrospective analysis of video data.

**Fig. 3:**
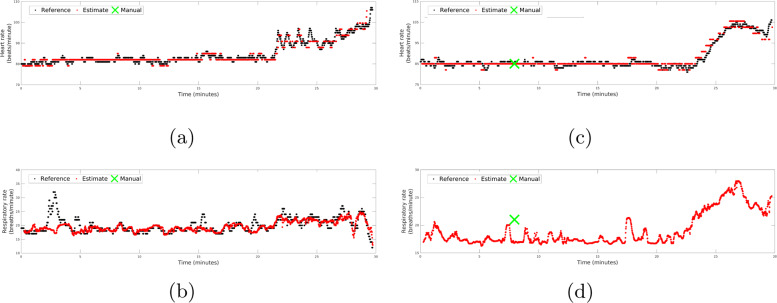
Reference and video camera-derived traces for two 30-min sample periods. Heart rate and respiratory rate values for the first period are shown in panels (**a**) and (**b**), respectively. Heart rate and respiratory rate values for the second period are shown in panels (**c**) and (**d**), respectively. Reference values were provided by the patient monitoring equipment (black), and manual nursing estimates (green). Fluctuations in heart rate associated with patient movement can be observed in (**a**) from *t* = 21 min to *t* = 25 min. The second 30-min sample (**c**, **d**) documents a period of deterioration from *t* = 20 min. The camera system produced frequent observations of both vital signs in during the episode of clinical deterioration. Only one manual respiratory rate was recorded during this period, as the impedance system was disconnected.

Figure 3 shows the camera-derived estimates compared against their corresponding gold-standard values for two 30-min sample periods. Heart rate values are shown in Fig. 3a, c. The respiratory rates measured in the same periods are shown in Fig. 3b, d. The manual measurements of the vital signs provided during routine clinical observation are also shown. For both segments, a good agreement is found between the reference signals and the video camera-derived estimates.

Episodes of short-term fluctuations in the vital signs, such as those observed in Fig. 3a from *t* = 21 min to *t* = 25 min, are known to be associated with bouts of patient activity. The transient changes in heart rate during this period were tracked by the video camera-derived estimates. Figure 3d presents a sample 30-min window during which no reference respiratory rate measurements were available. A manual measurement was taken at *t* = 8 min. The camera system produced frequent observations of both vital signs during the ensuing episode of acute deterioration (*t* = 20 min), including plausible estimates of respiratory rate. We observed that in such periods where wire-based respiratory monitoring was absent, the proposed algorithms produced estimates consistent with physiological status.

**Fig. 4:**
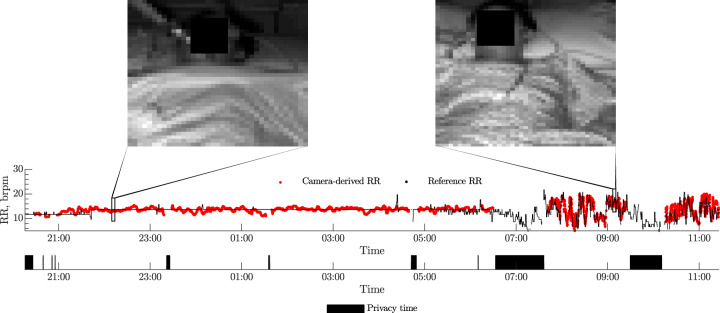
Respiratory monitoring of a patient over a 14-h period. The video camera-derived estimates (in red) are compared against the reference values provided by the bedside monitoring equipment (in black). Periods during which the privacy blind was drawn, and thus video monitoring was interrupted due an obstructed camera view of the patient, are shown in the bottom panel. Sample frames from the video camera recording during invasive respiratory support and after extubation are shown as inserts at *t =* 22:15 and *t =* 09:15.

Respiratory support plays an important role in care of patients with respiratory failure in the ICU. In Fig. 4, we show results for a patient undergoing respiratory support. The camera-derived estimates obtained from the regions of interest over the chest could provide a good approximation of the respiratory rate values of this patient under invasive respiratory support (14 breaths per minute). Following extubation, the spontaneous fluctuation of the respiratory rhythm in the ensuing period was accurately captured by the non-contact estimates produced from the analysis of video data, as shown in the same figure.

**Fig. 5:**
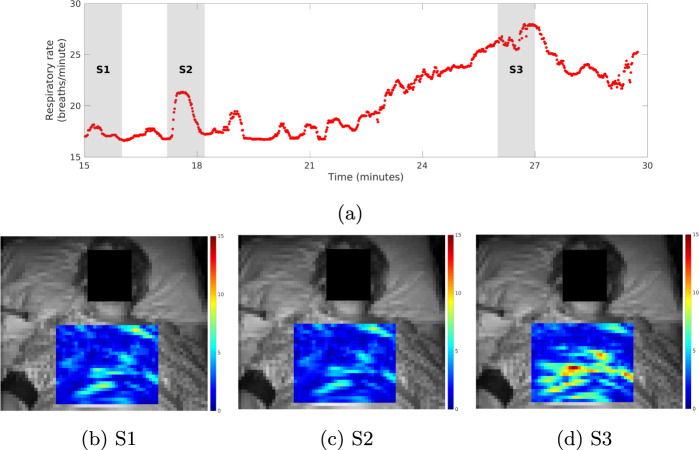
Visualisation of the local respiratory effort of a patient later diagnosed as having a ruptured diaphragm, as confirmed by chest CT, and a right-sided pneumothorax. (**a**) Respiratory rate estimated from the camera. Heat maps show the magnitude of the respiratory effort from regions on the chest during time stages (**b**) S1, (**c**) S2 and (**d**) S3. Each *super-pixel* in the heat maps was coloured according to the amplitude of the respiratory signal (in pixel units) computed from the 30 × 30 ROI centred on the *super-pixel*. The non-stationary respiratory rate was measured with a sustained increase in respiratory rate from *t* = 18 min to *t* = 27 min. A progression towards subdiaphragmatic left-lateral respiratory movements is observed as increased signal intensity over the lower left-side in the respiratory map for stage S3.

In Fig. 5 we show camera-derived results for a patient admitted to the ICU in whom a ruptured diaphragm and pneumothorax (confirmed by chest computer tomography) was diagnosed after the recording period. As a result of the ruptured diaphragm, the patient’s liver herniated into the chest. The diaphragm ruptured during the final 24 h of recording time, although it is not known exactly when this occurred. The non-stationary nature of the respiratory rhythm of this patient, marked by a sustained increase in the basal rate from *t* = 18 min to *t* = 27 min, was successfully tracked by the camera system throughout this period. A camera-derived map of local respiratory chest movements^33^ is also shown. As expected, this map shows areas of elevated respiratory amplitude over the patient’s lower chest, particularly along the line of the lower ribs. A lateralisation of the respiratory effort towards the left thorax was also observed in the later stage of this period.

## Discussion

We evaluated the feasibility of a non-contact monitoring system over clinically relevant time periods for critical care patients during post-operative care. We developed a novel system which successfully recorded video data from patients in the ICU. The design was acceptable to nursing staff^24^, complied with hospital safety criteria and provided reliable estimates of cardiorespiratory vital signs.

Previous work in non-contact vital-sign monitoring using video cameras has been conducted primarily under controlled conditions and with compliant healthy adults. One previous study of post-operative patients recorded short videos of patients following cardiac surgery for a mean duration of 28.6 ± 2.8 min per patient^48^. To our knowledge, ours is the first study of video-based non-contact monitoring study in an acute adult care setting for extended periods of time (over 12 h). The extended study period allowed us to assess the impact of routine clinical activities on the operation of the non-contact monitoring system, unlike shorter studies where clinical staff may tolerate short-term accommodations for the sake of good quality of the recorded data. While only three patients received over 24 h of monitoring, this was mostly due to unforeseen events, such as early discharge. Data were obtained both in the day-time and overnight, allowing algorithms to be tested under different light conditions and care protocols. Thus, our findings provide a good approximation of the performance of non-contact vital-sign monitoring systems in an acute clinical setting. Vital signs were estimated for the valid camera time, comprising a total of 119.8 h of respiratory rate monitoring (approximately 63.1% of the total number of hours of valid time) and a total of 103.0 h of heart rate monitoring (approximately 53.2% of the total number of hours of valid time). These figures reflect a difference in the number of time windows for which camera-derived cardiac and respiratory signals reached the quality threshold for rate estimation, and may be explained by the lower amplitude of the cardiac modulation of the camera signal.

As reported in Table 2, the vital-sign estimates derived from the video camera signals had high accuracy, with estimates within 2.5 beats/min and 2.4 breaths/min of the gold-standard reference values recorded by the standard patient monitoring equipment. Our error measures for heart rate monitoring are in line with those obtained using a camera monitoring system to record facial videos of patients during immediate recovery after cardiac surgery, which measured pulse rates with a bias of −3.7 ± 16.1 beats/min with respect to the ECG reference^48^. Hochhausen et al.^52^ used a long-wave infrared camera to record 28 post-operative patients (24 female, IQR 51–77 years) for short periods upon admission to a post-anaesthesia care unit and upon discharge from this unit. Estimates of the respiratory rate were obtained with a bias of 1.75 breaths/min and limits of agreement ranging from −2.74 to 1.75 breaths/min. In comparing these figures with those reported by us, it should be noted that Hochhausen et al. manually excluded the periods for which the ROI on the patient’s nose was not visible, or when patient movement was observed. Using a similar implementation of the algorithms described here, Villarroel et al. monitored patients undergoing haemodialysis treatment under the consistent ambient light of the kidney unit. They obtained an MAE of 2.8 beats/min for heart rate estimates for over 65% of the time the patient was stable. An MAE of 2.1 breaths/min was obtained for respiratory rate estimates over 69% of the time for which the reference signals were valid^32^. The gap in coverage time between the two scenarios may reflect differences between the two studies regarding the recording environment and the experimental protocol.

Two features in the distribution of respiratory rate values in Fig. 2 are worth noting. Most prior art in non-contact monitoring technology has relied on respiratory data available from healthy volunteers during metronome breathing. As a result, the distribution of measured rates available from volunteer studies is limited when compared to the one observed in this dataset. The large proportion of low rates in the [20, 30] breaths/min range is particularly relevant given that tachypnoea (RR > 20 breaths/min) is an established criterion for sepsis-evoked Systemic Inflammatory Response Syndrome (SIRS) in hospitalised patients^53^.

The non-contact camera system produced valid vital-sign estimates between routine manual observations in a busy ICU environment (see e.g. Fig. 3b). Once patients are discharged to a lower-acuity ward, where nurse observations are separated by several minutes or hours, the ability to track vital-sign changes between consecutive manual observations would be important in the context of early warning scores (EWSs). Considering that an elevated EWS score is an institutionally-defined criterion for escalating care, it is important that all times of increased EWS are noted^54^. The ability to provide continuous heart rate and respiratory rate estimates in addition to manual observations of the other vital signs may, therefore, be a useful addition to routine monitoring in the ward.

Although vital signs can be indicative of impending clinical deterioration, routine night-time monitoring adds to the already fragmented sleep of inpatients. Sleep disruption is prevalent among ICU patients^55^ and is associated with several negative outcomes, including elevated blood pressure and deliriu^56–58^. Therefore, it is important to consider strategies to reduce the burden of overnight monitoring. To our knowledge, ours is the first study to evaluate a non-contact video camera system in the night-time ICU environment. Primary outcomes were evaluated for the day-time period (102.0 h) and the night-time (131.5 h) period. Using an identical protocol for the recording and analysis of day-time and night-time data, comparable performance among the two periods has been demonstrated (Table 3). Lower error rates and higher correlation coefficient were observed for estimates of both vital signs during the night-time period. While further investigation is warranted to determine whether this is attributable to environmental factors, it is clear that the low-light setting did not prevent night-time video monitoring.

Video camera systems may provide diagnostic information in addition to vital-sign estimates. We revisited the case study of a patient who suffered a pneumothorax and ruptured diaphragm (Fig. 5), where we observed that alterations in the respiratory rhythm could be detected in the camera signal prior to diagnosis. Additionally, the processing of the video camera data revealed asymmetries between regional breathing movements over the chest and thorax. These findings highlight the potential role of video camera monitors not only as a viable method of monitoring the respiratory rate but also of providing a functional assessment through a contextual understanding of morphology. This is made possible through the access to visual cues not available to conventional monitors, or even wearable wireless monitoring systems. Further research will aim to show that the features in the video signal can similarly capture key haemodynamic events in this patient group, such as the onset of clinically significant hypovolaemia or the occurrence of mottled skin (see ISRCTN study reference^59^).

Some study limitations need to be addressed. Data were collected at a single ICU in a teaching hospital located in Oxford, UK. Thus, our results may neither be generalisable to other ICUs in the UK, nor to other healthcare systems, where clinical protocols for ICU admission, surveillance, and discharge of surgical patients may differ. A demonstration of feasibility on ICU does not necessarily translate to lower-acuity units, where patients are usually not tethered to a patient monitor. A wide-angle lens system is needed for day-time monitoring as patients can ambulate freely, as has been achieved in mental health settings by a system developed in parallel with the ICU system^60^. The mean age of the patient cohort was 62.2 years (Table 1), which may limit our findings to older populations. Similarly, the study sample did not include participants with skin type ⩾ IV on the Fitzpatrick scale. The performance of heart rate estimation algorithms is known to be negatively affected by darker skin tones and thus further validation is required on a more diverse cohort. The study was not adequately powered to assess the impact of patient position on the accuracy and coverage of video camera-based monitoring. Finally, the use of the privacy blind to obscure the view of the patient from the camera did overlap with some noteworthy events. Several periods of tachycardia were associated with moments where the privacy blind was drawn, precluding non-contact monitoring throughout the associated interventions. The accuracy of camera algorithms over a range of heart rate values wider than that observed in the study cohort has been shown in previous studies in the adult^33^ and the neonatal^32^ population. All data analysis was carried out offline and did not affect care in real-time.

These limitations notwithstanding, our results demonstrate the potential of non-contact continuous vital-sign monitoring over clinically relevant time periods to allow real-time detection of physiological deterioration.

## Methods

This was an observational study designed to evaluate the feasibility of introducing a non-contact monitoring system into the adult ICU of the Churchill Hospital, in Oxford, United Kingdom. The study was part of a research initiative of the Oxford University Hospitals National Health Service (NHS) Foundation Trust and the Oxford Biomedical Research Centre. The research was compliant with the relevant government regulations. Ethical approval was granted by the Wales Research Ethics Committee 5 (Bangor) under reference number 16/WA/0024. The study was registered in the NIHR Clinical Research Network (CRN) Portfolio under CPMS ID 30402 (IRAS ID 182738).

### Study design and protocol

Elective post-surgical patients with a planned post-operative admission to the Oxford Churchill Hospital ICU were considered for inclusion. We aimed to recruit 25 patients. Eligible patients were recruited from the pre-operative assessment clinics servicing the following specialities: maxillofacial surgery, gastrointestinal surgery, hepatobiliary surgery, renal transplant, pancreatic transplant, urology, and gynaecology. Candidate patients were screened by members of the clinical team against inclusion/exclusion criteria, and eligible patients were approached for informed consent. The exclusion criteria in Table 4 were considered.

**Table 4:** Participant exclusion criteria.

Exclusion criteria
Patients under the age of 18
Patients whose anatomy precludes the use of the required monitoring
Patients who were judged to lack capacity at the time of interest, e.g. due to an illness, severe learning disability,
intoxication caused by a drug, or otherwise
Patients unable to understand written English and for whom no translator could be found

Recording was initiated as soon as practically possible after admission to the ICU following surgery. Each patient was monitored continuously for up to 48 h, or until discharge from the ICU. For the duration of the study, concurrent video and physiological data collected by the patient monitor system used as part of routine care were recorded. Equipment was chosen such that it would not affect the experience of the patient or clinical staff. Vital sign estimates from the non-contact monitoring software were computed retrospectively and were not available to clinical staff during the study.

A privacy blind (an opaque sheet of plastic attached to a hinged fitting of the video camera) was included. This blind could be used to obscure the view of the patient from the video camera and thus prevent the recording during dignity-compromising situations (such as routine hygiene). Patients could ask for the blind to be drawn at any time. Patients, their visitors, or staff members could also ask for the equipment to be turned off for any reason throughout the study.

Select clinical information was documented by clinical research staff on paper-based case report forms: patient demographics, admission category, recording start and end times, APACHE II score^61^, Richmond Agitation-Sedation Scale (RASS), Fitzpatrick score, and free-text clinical notes. The Fitzpatrick Scale is a numerical skin classification system, from Type I (pale white, always burns, never tans) to Type VI (darkest brown, never burns, never tans)^49^.

#### Instrumentation

**Fig. 6:**
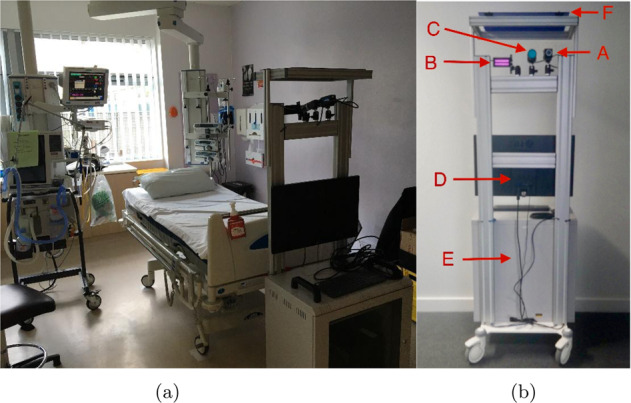
Monitoring apparatus in the Churchill Hospital ICU in Oxford, United Kingdom. **a** Patient monitor at the bedside and the non-contact vital-sign monitoring equipment at the end a patient’s bed. **b** Set-up of the trolley containing the non-contact vital-sign monitoring equipment. The components are shown in their respective positions—(A) optical camera; (B) infrared LED source; (C) thermal camera; (D) interface equipment; (E) workstation (in cabinet); (F) privacy blind (folded up).

The monitoring equipment is shown in Fig. 6a. As per the standard of care, vital-sign monitoring was provided using a IntelliVue^®^ MP70 patient monitor (Philips, Eindhoven, Netherlands). Using proprietary software, reference values of HR and RR were derived at 1-s increments. HR was derived from the single-lead ECG (sampled at 250 Hz), while RR was derived from the bipolar IP signal collected (at 62.5 Hz) using the same set of electrodes. Pulse rate was obtained from the photoplethysmogram (PPG) signal acquired by a pulse oximetry Philips ^®^ M1941A finger probe (Philips, Eindhoven, Netherlands) and sampled at 125 Hz.

Patients were monitored using a 3.2 megapixel Point Grey Research Grasshopper3^®^ GS3-U3-32S4M-C monochrome camera (FLIR Instruments, Oregon, USA). A non-polarised lens with a 12.5 mm fixed focal length and an aperture range of f/1.4-16 was attached (Goyo Optical^®^ GMHR412514MCN, Saitama, Japan), as well as an infrared-pass filter (Schneider Kreuznach^®^ 098, Germany). The imaging device was set to acquire raw uncompressed 8-bit image data at a resolution of 1024 × 768 pixels and a nominal sampling rate of 100 Hz. Illumination was provided using a Metaphase^®^ FL201-IRN-24 850 nm infrared illuminator (Metaphase, Bristol, Pennsilvania, USA). Since 850 nm is outside the visible range, the illuminator did not emit any noticeable glow light. The infrared illuminator powered by a Metaphase^®^ ULC-2 LED controller (Metaphase, Bristol, Pennsylvania, USA) connected to a Gardasoft^®^ CC320 sequence controller (Gardasoft, Cambridge, UK) was strobed at 50 Hz to illuminate alternate video frames.

The equipment was designed to be non-disruptive to standard care. A medical-grade hospital trolley was used as the basis of the non-contact monitoring system. Patients in the ICU are accommodated on any available bed, and so equipment needed to be mobile rather than installed at one particular bed space. The video camera devices and the infrared illuminator were mounted on a hospital trolley positioned at the end of the patient’s bed, as shown in Fig. 6a. The trolley was easily movable so it would not impede access to the patient. As is usual practice, patients had access to a chair beside the bed, on which they would occasionally sit. Data were captured for both locations.

All recorded video and physiological data were relayed to an 8 TB Hewlett-Packard (HP) workstation. The workstation was replaced every 12 h with an identical one, due to the large storage requirements (approximately 6.7 GB per minute for the digital video data). All video data were encrypted using industry standard methods.

### Data analysis

The video data were reviewed by research nurses and any privacy-compromising sections of data were deleted. Furthermore, video data containing individuals who had not been consented (e.g. family members, nurses, etc.) were also removed by research nurses using a bounding box around the patient. In instances where the patient and the non-consenting individual could not be separated in the video frame (e.g. because they obscured part of the view of the patient), the entire frame was deleted.

Prior to vital-sign estimation, the collected video data were analysed so that time periods for which the privacy blind was drawn, or nurse-reviewed frames were deleted, were discarded from the valid time available for vital-sign estimation. The methods used to obtain non-contact estimates of vital signs during valid time were subsequently applied.

Cardiac-synchronous and respiratory waveforms were extracted from the video using image processing algorithms that analyse several regions of interest across the available frame areas, such as the patient’s face or chest. Our pipeline for video processing has been reviewed in previous studies^31–33^ and so it is described here in brief.

To assess the feasibility of non-contact monitoring, the video camera-derived estimates of both vital signals were assessed against the gold-standard values provided by a clinical-grade patient monitor from analysis of the physiological signals acquired at the bedside. We assessed performance in terms of error measures, Pearson correlation, and estimated time, expressed as a percentage of total and valid recording times.

### Reference physiological values

The measurement of the physiological signals of hospital patients with contact devices is subject to periods of artefactual noise due to factors such as patient movement, or poor probe placement. For this reason, some processing of the heart rate and respiratory rate values obtained from the patient monitor is needed prior to their use as references. A gold-standard reference of heart rate was derived based on the redundancy of vital-sign measurements provided by the multiple sources available at the bedside. The results of this procedure are provided as a supplemental material to this submission.

The performance of the proposed vital-sign estimation system was assessed over those periods for which this gold-standard was available, referred to as valid time.

### Heart rate estimation

A signal processing pipeline for the estimation of heart rate validated in clinical studies^31–33^ was adopted. A PPGi signal was extracted from a grid of ROIs defined over the available frame area. We then used tested algorithms to assess the quality of the cardiac pulses found in each PPGi signal. For each time window, a candidate HR value was estimated from the PPGi signal extracted from each sub-region using both time-domain and frequency-domain algorithms. A definitive heart rate estimate for that time window was computed by combining the individual estimates from each of the previous methods using a data fusion technique. Finally, the time window was shifted by a number of PPGi samples corresponding to a time shift of 1 s, and these steps were repeated for the new window. Therefore, heart rate estimates were reported every second. A window length of 15 s was used.

#### Extraction of PPGi signal

A region of interest ROI_*S*_ over the available frame area was obtained for every image frame. A total of *N* = 81 sub-regions of ROI_*S*_ organised in a 9 × 9 grid over this ROI were defined. In the few instances where the bounding box selected by the research nurses was limited to small pixel area, a 3 × 3 grid was used instead. The average pixel intensity over each sub-region was subsequently computed for every image frame. Several step changes can occur during video recording. A Bayesian change point detection algorithm was applied to 30-s running windows with a 5-s overlap^62^. The resulting time series {ROI_*S*,1_, . . . , ROI_*S*,*N*_} is known to contain signal components correlated with the cardiac frequency as well as artefactual components due to noise sources, such as motion and changes in ambient illumination. To attenuate spectral components outside the range of physiological heart values, from 36 to 210 beats/min^63^, the PPGi signals were linearly detrended and a 50^*th*^ order finite-impulse response (FIR) band-pass filter with cut-off frequencies of 0.6 Hz and 3.5 Hz was applied.

The filtered ${\{{\rm{RO}}{{\rm{I}}}_{S,i}\}}_{i = 1,...,N}$ waveforms were then subdivided into 15-s running windows with a time shift between consecutive windows of 1 s. For a given time window, a set *N* filtered waveforms, i.e. one per each of the *N* sub-regions, were available.

#### Estimation of heart rate from PPGi

The PPGi signals extracted for each window were used as inputs to a heart rate estimation process reported in earlier studies^32,33^. In summary, a peak and onset detection algorithm based on Zong et al.^64^ and extended by Villarroel et al.^32^ was applied to identify salient points in the PPGi signal. Accurate peak and onset detection allowed beat-by-beat assessment of the pulsatile signal quality using a signal quality index (SQI) by comparing each beat with a running template using several metrics that measure: amplitude differences, the occurrence of step changes, beat pulses outside the expected physiological range and a similarity metric based on a multi-scale Dynamic Time Warping (DTW) algorithm^65^.

Heart rate was computed using an autoregressive spectral analysis^31^. Once candidate heart rate estimates had been computed for every 15-s window (one from each sub-region), the heart rate estimate for a given window was computed as the median of all the heart rate estimates from each colour channel from the ROI_*S*_ that had an associated SQI greater than 0.8. As in Villarroel et al., a Kalman filter was applied to fuse these estimates over time based on their signal quality^65^, thus reducing the effects of transient changes in pixel intensity. Fused heart rate estimates were reported every second.

### Respiratory rate estimation

During normal respiration, the contraction and relaxation of the abdominal and intercostal muscles causes the volume of the chest cavity to increase or decrease. The volumetric changes with airflow cause movement of the body, principally from regions around the thorax and diaphragm, which can be recorded by a video camera from areas of exposed skin but also from those covered by tight-fitting or highly patterned clothing.

This phenomenon has previously been exploited for the non-contact continuous monitoring of respiration. Several methods have been proposed to extract respiratory signal from the breathing-related movements of the thorax^66–68^, thoracoabdominal area^66,69,70^, or face area^71^. Previous studies have shown that respiratory signals can be detected as modulations to the PPGi signal extracted from the subject’s skin areas^32,39,72^.

In this work, respiratory signals were acquired by measuring the respiratory-induced intensity changes in multiple ROIs across the available frame area. Respiratory rate was then computed by using data fusion algorithms from the analysis of the signals extracted from the ROIs. This methodology is described below. The pipeline adopted for both the extraction of the respiratory signal and the estimation of respiratory rate have been validated in previous clinical trials^32,33^.

#### Extraction of respiratory signal

A second region of interest (ROI_*T*_) over the available frame area was obtained for every image frame. A total of *M* = 9 sub-regions organised in a 9 × 9 grid over ROI_*T*_ were defined.

For every frame, {ROI_*T*,1_, . . . , ROI_*T*,*M*_} signals were extracted by averaging the pixel intensity signal over each sub-region. The *M* signals extracted are analogous to the {ROI_*S*,1_, . . . , ROI_*S*,*N*_} time series extracted in the previous sections for heart rate estimation. The extracted signals contain both cardiac and respiratory information. It has been shown that the relative weight of the respiratory-to-cardiac related pulsatile components in the extracted signal depends to a great extent on the size of the region of interest^31^. In addition, the signals extracted are often contaminated by baseline drifts and high-frequency noise^70^. To remove these artefacts, each *M* signal was detrended and filtered using a cascade of a 8th order high-pass IIR filter with a cut-off frequency at 0.1 Hz and an 2nd order low-pass IIR filter with a cut-off frequency at 0.8 Hz. These filters encompass respiratory rates values in the range of 6–48 breaths/min. Once the {ROI_*T*,1_, . . . , ROI_*T*,*M*_} were processed, the resulting waveform was subdivided into 30-s running windows with a step of 5 s between consecutive windows. Similar to the methodology adopted for the extraction of PPGi signals, the {Respi_*i*_} respiratory waveforms obtained were used as inputs to a respiration rate estimation block.

#### Estimation of respiratory rate

Our general approach to deriving respiratory rate for each time window is as follows. The peaks and onsets were detected using an adaptive algorithm previously described^33^. For every window, a candidate respiratory rate value was computed for each of the *Respi* using the autorregressive methods described for heart rate estimation.

Respiration has been shown to be more prominent in quiet research participants with minimal body motion. Thus, for each time window and for each of the respiratory signal extracted, signal quality indices were computed as detailed in the previous work^33^. Once both the candidate respiratory rate and signal quality index had been estimated for all respiratory signals, a data fusion technique based on multiple Kalman filters (as for heart rate estimation) was applied to combine the multiple respiratory estimates from the same time window and produce a final respiratory rate for each time window. Fused respiratory rate estimates were reported every 5 s.

### Localised respiration mapping

Spatial maps of respiratory signal amplitude were reported for a patient with abnormal respiratory physiology using methods developed in^33^. Each *super-pixel* in the maps was coloured according to the amplitude of the respiratory signal computed from the 30 × 30 ROI centred on the *super-pixel*. The resulting respiratory maps were smoothed using a 2D Gaussian filter with a filter size of 3 × 3 (*super-pixels*) and a kernel width of 0.5 such that the filter only operated on neighbouring *super-pixels*.

### Reporting summary

Further information on research design is available in the Nature Research Reporting Summary linked to this article.

## Supplementary information

Reporting Summary

Supplementary Information

## Data Availability

To protect the privacy of the patients involved in the study, the datasets analysed in this study are not available under the granted research ethics application.

## Code availability

The codes used in the study are proprietary, and available only with the permission of the licensors.
