# Supplementary material for: Non-contact physiological monitoring of post-operative patients in the intensive care unit
Source: NPJ Digit Med. 2022 Jan 13;5:4. doi: 10.1038/s41746-021-00543-z (PMC8758749; doi:10.1038/s41746-021-00543-z)
Supplement: Supplementary file 1 — Reporting Summary [file 41746_2021_543_MOESM1_ESM.pdf]

nature portfolio

Corresponding author(s): Dr Joao Jorge

Last updated by author(s): 21-11-17

Reporting Summary

Nature Portfolio wishes to improve the reproducibility of the work that we publish. This form provides structure for consistency and transparency in reporting. For further information on Nature Portfolio policies, see our Editorial Policies and the Editorial Policy Checklist.

Statistics

For all statistical analyses, confirm that the following items are present in the figure legend, table legend, main text, or Methods section.

n/a	Confirmed	
☐	☒	The exact sample size (*n*) for each experimental group/condition, given as a discrete number and unit of measurement
☐	☒	A statement on whether measurements were taken from distinct samples or whether the same sample was measured repeatedly
☐	☒	The statistical test(s) used AND whether they are one- or two-sided<br>*Only common tests should be described solely by name; describe more complex techniques in the Methods section.*
☒	☐	A description of all covariates tested
☐	☒	A description of any assumptions or corrections, such as tests of normality and adjustment for multiple comparisons
☐	☒	A full description of the statistical parameters including central tendency (e.g. means) or other basic estimates (e.g. regression coefficient) AND variation (e.g. standard deviation) or associated estimates of uncertainty (e.g. confidence intervals)
☒	☐	For null hypothesis testing, the test statistic (e.g. *F*, *t*, *r*) with confidence intervals, effect sizes, degrees of freedom and *P* value noted<br>*Give P values as exact values whenever suitable.*
☒	☐	For Bayesian analysis, information on the choice of priors and Markov chain Monte Carlo settings
☒	☐	For hierarchical and complex designs, identification of the appropriate level for tests and full reporting of outcomes
☐	☒	Estimates of effect sizes (e.g. Cohen's *d*, Pearson's *r*), indicating how they were calculated

Our web collection on statistics for biologists contains articles on many of the points above.

Software and code

Policy information about availability of computer code

Data collection	To record the data generated by the Philips patient monitor, we used the ixTrend software (IxellenceGmbH, Germany). The real-time software to record videos was designed and developed by the authors.
Data analysis	The software for video processing has been published in an earlier publication available at https://www.nature.com/articles/41746-019-0199-5. The signal processing algorithms were developed in Matlab 2020b.

For manuscripts utilizing custom algorithms or software that are central to the research but not yet described in published literature, software must be made available to editors and reviewers. We strongly encourage code deposition in a community repository (e.g. GitHub). See the Nature Portfolio guidelines for submitting code & software for further information.

Data

Policy information about availability of data

All manuscripts must include a data availability statement. This statement should provide the following information, where applicable:

- Accession codes, unique identifiers, or web links for publicly available datasets
- A description of any restrictions on data availability
- For clinical datasets or third party data, please ensure that the statement adheres to our policy

The datasets available during the study are not available for access due to the sensitive and identifiable nature of the data, patient consent and restrictions in the ethics protocol to protect the privacy of patients involved in the study.

Field-specific reporting

Please select the one below that is the best fit for your research. If you are not sure, read the appropriate sections before making your selection.

☒ Life sciences ☐ Behavioural & social sciences ☐ Ecological, evolutionary & environmental sciences

For a reference copy of the document with all sections, see nature.com/documents/nr-reporting-summary-flat.pdf

Life sciences study design

All studies must disclose on these points even when the disclosure is negative.

Sample size	A pilot cohort of 30 patients was analysed.
Data exclusions	Patients under the age of 18; Patients whose anatomy precludes the use of the required monitoring; Patients who were judged to lack capacity at the time of interest, e.g. due to an illness, learning disability, or intoxication caused by a drug, or otherwise; Patients unable to understand written English and for whom no translator could be found.
Replication	N/A. this was an exploratory study to determine if it was possible to estimate the heart rate and respiratory rate of post-operative patients admitted to the Adult Intensive Care Unit.
Randomization	N/A
Blinding	The analyst team was blinded to patient clinical and demographic data.

Reporting for specific materials, systems and methods

We require information from authors about some types of materials, experimental systems and methods used in many studies. Here, indicate whether each material, system or method listed is relevant to your study. If you are not sure if a list item applies to your research, read the appropriate section before selecting a response.

Materials & experimental systems

n/a	Involved in the study	
☒	☐	Antibodies
☒	☐	Eukaryotic cell lines
☒	☐	Palaeontology and archaeology
☒	☐	Animals and other organisms
☐	☒	Human research participants
☐	☒	Clinical data
☒	☐	Dual use research of concern

Methods

n/a	Involved in the study	
☒	☐	ChIP-seq
☒	☐	Flow cytometry
☒	☐	MRI-based neuroimaging

Human research participants

Policy information about studies involving human research participants

Population characteristics	Patients with a planned post-operative admission to the Oxford Churchill Hospital ICU were considered for inclusion. Eligible patients were recruited from the pre-operative assessment clinics servicing the following specialities: maxillo-facial surgery, gastrointestinal surgery, hepatobiliary surgery, renal transplant, pancreatic transplant, urology, and gynaecology.
Recruitment	Eligible patients were recruited from the pre-operative assessment clinics. Candidate patients were screened by members of the clinical team against inclusion/exclusion criteria, and eligible patients were approached for informed consent.
Ethics oversight	The research was compliant with the relevant government regulations. Ethical approval was granted by the Wales Research Ethics Committee 5 (Bangor) under reference number 16/WA/0024.

Note that full information on the approval of the study protocol must also be provided in the manuscript.

Clinical data

Policy information about clinical studies

All manuscripts should comply with the ICMJE guidelines for publication of clinical research and a completed CONSORT checklist must be included with all submissions.

Clinical trial registration	NIHR Clinical Research Network (CRN) Portfolio under CPMS ID 30402 (IRAS ID 182738)

Study protocol	https://public-odp.nihr.ac.uk/QvAJAXZfc/opendoc.htm?document=CRNCC Users%2FFind%20A%20Clinical%20Research%20Study.qvw&sheet=SH01&bookmark=Document\BM02&select=LB01,=StudyID=30402
Data collection	Patients monitored in the adult ICU of the Churchill Hospital, in Oxford, United Kingdom over the course of one year.
Outcomes	In this study we will establish the feasibility of non-contact patient monitoring for extended periods in an acute clinical setting. We will evaluate the amount of usable data that can be collected from the thermal and digital video cameras, and compare the derived vital signs with data obtained simultaneously through traditional contact sensors.
