# Supplementary material for: Non-contact physiological monitoring of post-operative patients in the intensive care unit
Source: NPJ Digit Med. 2022 Jan 13;5:4. doi: 10.1038/s41746-021-00543-z (PMC8758749; doi:10.1038/s41746-021-00543-z)
Supplement: Supplementary file 2 — Supplementary Information [file 41746_2021_543_MOESM2_ESM.pdf]

# Non-contact physiological monitoring of post-operative patients in the Intensive Care Unit

**João Jorge[1,2,+,*], Mauricio Villarroel[1,2,+], Hamish Tomlinson[1], Oliver Gibson[3], Julie L Darbyshire[4], Jody Ede[2,4], Mirae Harford[2,4], John Duncan Young[4], Lionel Tarassenko[1,2], and Peter Watkinson[2,4,5]**

[1]Institute of Biomedical Engineering, Department of Engineering Science, University of Oxford, UK.
[2]NIHR Biomedical Research Centre, Oxford, UK.
[3]Oxehealth Ltd., The Oxford Science Park, Magdalen Centre North, UK.
[4]Kadoorie Centre for Critical Care Research and Education, Nuffield Department of Clinical Neurosciences, University of Oxford, UK.
[5]Oxford University Hospitals NHS Trust, UK.
[+]these authors contributed equally to this work, names are listed in alphabetical order.
[*]e-mail at:joao.jorge@eng.ox.ac.uk.

## ABSTRACT

Supplementary materials 1 and 2.

## Supplementary material 1

### Computation of valid time

The vital-sign waveforms provided by the patient monitor include periods of missing or artefactual values as a result of patient movement or probe disconnection. The assessment of their quality prior to their use as a reference is, therefore, of fundamental importance so that performance assessment is only attempted in time epochs for which the recorded signals communicate reliable representations of the physiological rates (the valid time). As described in the main body of this submission, good-quality monitor HR and RR values were identified. Gold-standard reference rates were computed and used to assess the performance of video-derived estimates in our submission in lieu of the original rates provided by the patient monitor. This supplementary material provides the protocol used for quality assessment and a summary of results.

#### *Reference respiratory rate*

ICU patients are typically single-monitored for respiratory rate through impedance pneumography. Although IP is a convenient method in this setting as patients are already monitored by electrocardiography, it is notoriously prone to inaccurate readings due to a number of factors including poor electrode placement, motion artefact and high sensitivity to non-breathing thoracic movements[1]. To exclude implausible estimates, a physiological filter was applied whereby respiratory rate estimates outside the plausible range for adults (*i.e.* 5 to 50 breaths per minute) were discarded. Outliers were removed using a 60-second median filter. Supplementary Figure 1a shows the profile of the re-derived gold-standard reference respiratory rate against the original respiratory estimates for a sample 45-minute segment extracted from a study session in the main submission.

#### *Reference heart rate*

Two reference heart rate signals were provided by the patient monitor: the heart rate derived by this monitor from the single-lead ECG waveform, and pulse rate derived from the finger pulse oximeter, both sampled at a rate of 1 Hz. The benefit of having

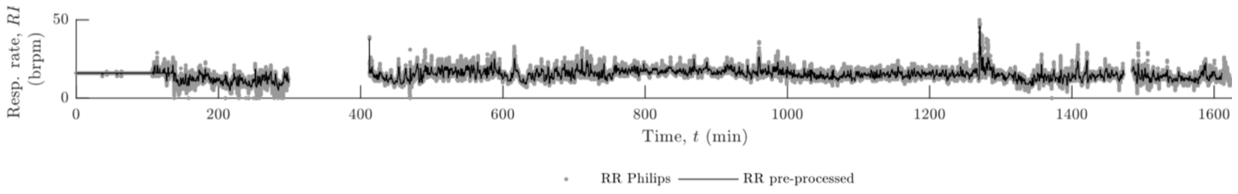


**(a)**

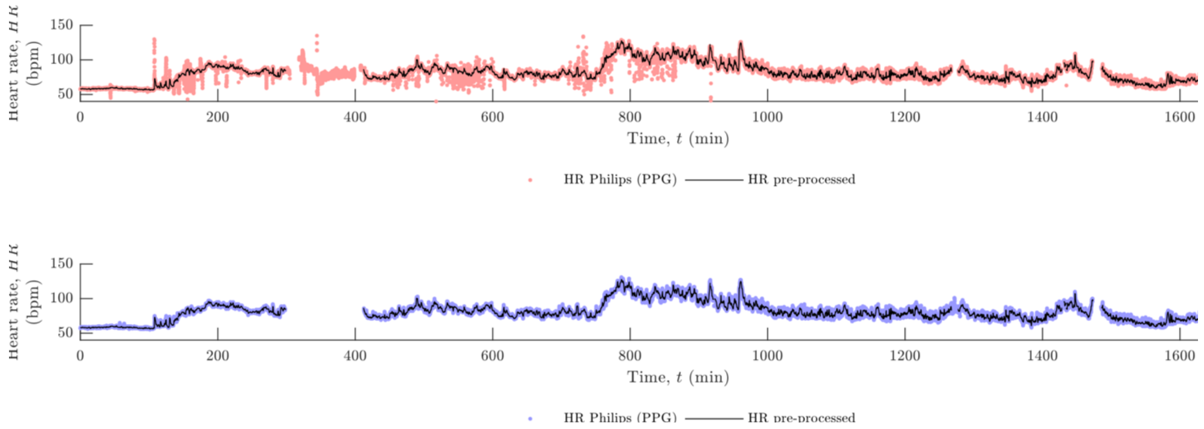


**(b)**

**Supplementary Figure 1.** Gold-standard reference rates computed for a sample segment. (**a**) Respiratory estimates derived by the patient monitor (in grey) and the new reference (in black). (**b**) Heart rate estimates from the ECG (in red) and PPG (in blue), and the new reference (in black).

two measurement devices is that the agreement between the two can imply that the heart rate measurements are of good quality. Conversely, discrepancies between the two can reveal periods during which the signal was severely corrupted by noise or motion artefacts.

A gold-standard reference heart rate $HR_{ref}$ was computed as the mean between the ECG-derived heart rate estimates and the PPG-derived pulse rate estimates for those time points for which the difference between these values did not exceed 5 beats per minute[2]. Only those values within the physiological range for adults, *i.e.* 40 to 120 beats per minute (bpm), were considered. Outliers were removed using a 60-second median filter. Supplementary Figure 1 presents the gold-standard reference values obtained after this pre-processing for a sample recording session, and compares these computed rates against the original the heart rate and pulse rate estimates produced by the patient monitor. While a good agreement can be found between both sources of estimates for most of this session, the pre-processing step introduced here allowed for the exclusion of outliers (*e.g.* around $t = 100$ min or $t = 500$ min from the gold-standard reference.

Supplementary Table 1 details the monitoring time for each study participant. Of the 233.5 hours for which simultaneous video and monitor data were available, a gold-standard reference heart rate was obtained for 224.1 hours (96.0 %). A gold-standard reference respiratory rate could be obtained for 218.8 hours (93.7 %). Periods for which the privacy blind was in use (private time) are also shown. Vital sign estimation was attempted for the remaining time, *i.e.* valid time. Thus, the valid data comprised the subset of video data for which the privacy blind was not in use and a good quality reference (of heart rate or respiratory rate) was available for comparison. The performance of camera-derived estimates is presented in Table 2 in the main submission.

**Supplementary Table 1.** Summary of reference signal quality for all recording sessions.

| Patient | Total recording time (hours) | Private time (hours) | Heart rate | | Respiratory rate | |
|---|---|---|---|---|---|---|
| | | | Valid reference (hours, %)[1] | Valid data (hours, %)[1] | Valid reference (hours, %)[1] | Valid data (hours, %)[1] |
| 1 | 15.2 h | 2.2 h | 14.5 h, 95.2% | 12.4 h, 81.9% | 10.5 h, 69.0% | 9.2 h, 60.5% |
| 2 | 27.1 h | 4.9 h | 24.8 h, 91.4% | 20.0 h, 73.7% | 25.9 h, 95.7% | 21.1 h, 77.7% |
| 3 | 5.8 h | 2.1 h | 5.8 h, 99.8% | 3.8 h, 64.7% | 2.4 h, 40.7% | 1.7 h, 29.8% |
| 4 | 18.1 h | 3.4 h | 18.1 h, 99.7% | 14.7 h, 81.1% | 20.0 h, 110.6% | 16.4 h, 90.8% |
| 5 | 1.3 h | 0.2 h | 1.0 h, 75.7% | 0.9 h, 65.7% | 1.3 h, 99.8% | 1.1 h, 85.8% |
| 6 | 15.9 h | 0.6 h | 15.8 h, 99.7% | 15.3 h, 96.1% | 11.5 h, 72.4% | 11.3 h, 71.4% |
| 7 | 5.5 h | 0.0 h | 4.3 h, 77.8% | 4.3 h, 77.8% | 5.7 h, 102.9% | 5.6 h, 102.7% |
| 8 | 16.0 h | 2.6 h | 15.9 h, 99.7% | 13.3 h, 83.5% | 16.1 h, 100.6% | 13.5 h, 84.3% |
| 9 | 21.1 h | 0.7 h | 21.0 h, 99.8% | 20.3 h, 96.4% | 16.1 h, 76.5% | 15.4 h, 73.2% |
| 10 | 26.8 h | 4.4 h | 26.8 h, 100.0% | 22.4 h, 83.5% | 25.2 h, 94.1% | 21.3 h, 79.5% |
| 11 | 30.4 h | 4.5 h | 30.4 h, 100.0% | 25.9 h, 85.2% | 28.0 h, 92.1% | 23.6 h, 77.7% |
| 12 | 19.3 h | 2.9 h | 15.8 h, 81.7% | 13.3 h, 68.8% | 20.2 h, 104.6% | 17.2 h, 89.1% |
| 13 | 10.1 h | 2.0 h | 10.1 h, 100.0% | 8.1 h, 80.5% | 9.3 h, 92.2% | 7.4 h, 73.8% |
| 14 | 14.8 h | 0.3 h | 14.7 h, 99.8% | 14.4 h, 97.7% | 14.2 h, 96.3% | 13.9 h, 93.9% |
| 15 | 6.2 h | 0.4 h | 5.1 h, 82.6% | 4.7 h, 75.5% | 7.3 h, 119.1% | 6.9 h, 112.0% |
| Overall | 233.5 h | 31.2 h | 224.1 h, 96.0% | 193.7 h, 82.9% | 218.8 h, 93.7% | 189.7 h, 81.3% |

[1] Percentage with respect to the total recording time.

### Solving the problem of inter-device time delays

The task of recording the time instants at which measurements are taken is left to the clock inside each device, even before this data is relayed to other devices, such as a workstation or a datalogger. This gives rise to a delay between the raw signals collected by different devices (*e.g.* PPG from an oxygen saturation finger, or ECG signals from chest electrodes). Such a delay also applies to the parameters derived at device level, *e.g.* pulse rate derived from the PPG signal by the finger probe and the heart rate derived from the ECG signal by the ECG system). This inter-device delay needs to be measured and compensated for before signals from different sources can be compared. Prior to error analysis, camera-derived and monitor-derived vital sign estimates were time-aligned in the manner described in[3] using the cross-correlation function between the two signals for a range of plausible time shifts. The time shift corresponding to the peak cross-correlation value for that session was assumed to correspond to the time lag between the two sources.

## Supplementary material 2

### Day-time and night-time estimation

Figures 1 and 2 in the main submission show agreggate results regarding the agreement between the reference vital sign values (computed from the physiological monitor) and the video camera estimates for the entire recording period and for the 15 patients. This supporting material provides a breakdown of these two figures for the two periods of interest: day-time (08:00 to 19:59) and night-time (20:00 to 07:59).

Table 3 in the main submission summarises the performance of video camera-based estimation of vital signs each of the two periods.

### *Day-time period*

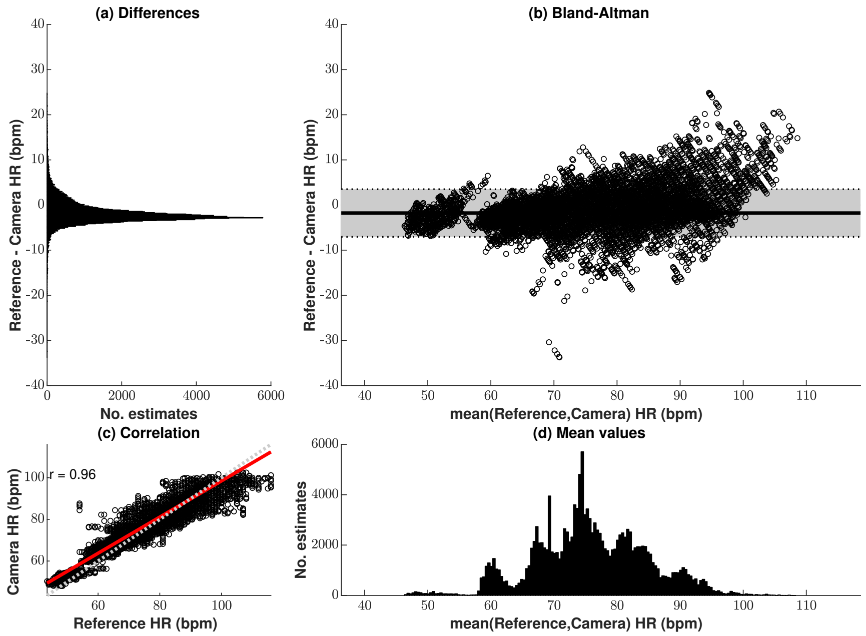


**Supplementary Figure 2.** Agreement between the reference heart rate values (computed from the ECG and PPG) and the camera estimates during day-time hours, comprising a total estimated time of approximately 42.6 hours.

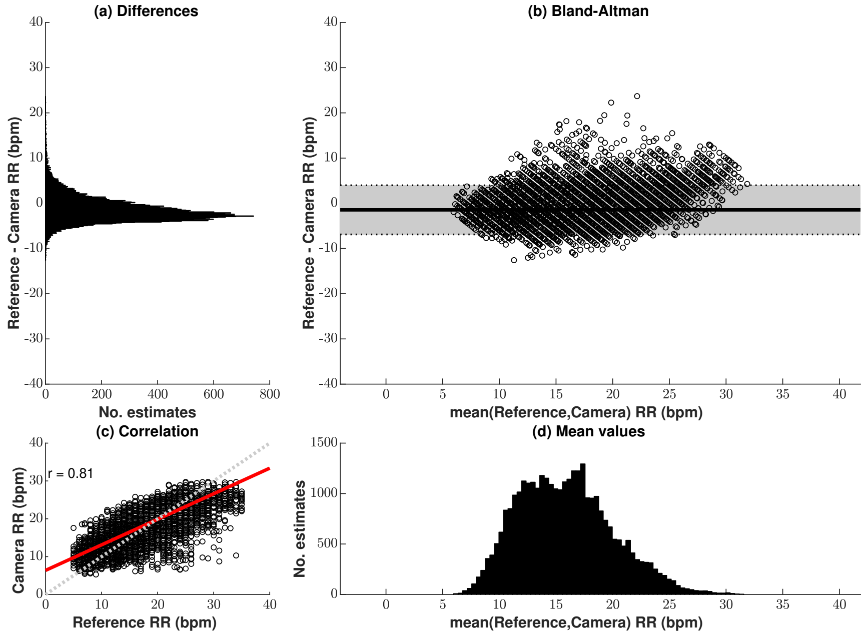


**Supplementary Figure 3.** Agreement between the reference respiratory rate values (computed from the IP) and the camera estimates during day-time hours, comprising a total estimated time of approximately 45.1 hours.

***Night-time period***

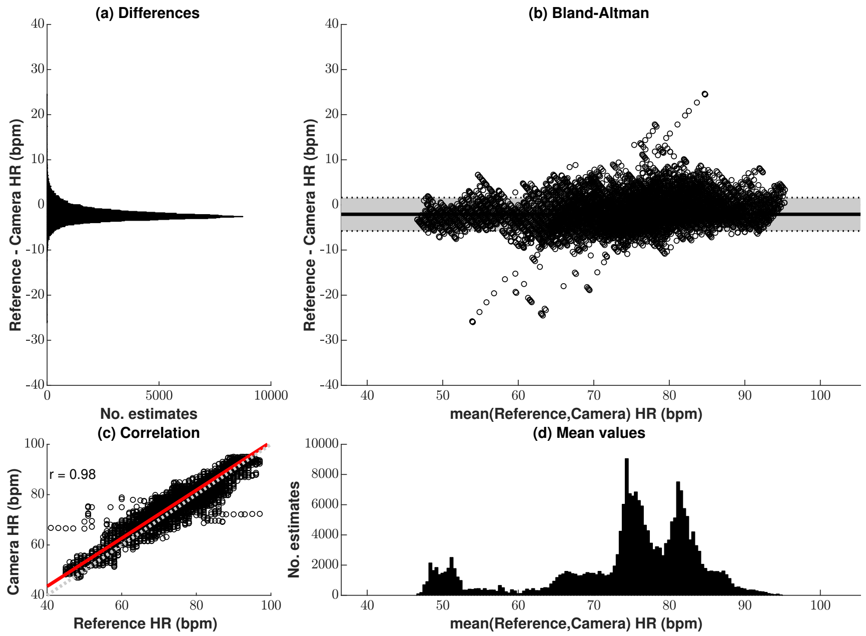


**Supplementary Figure 4.** Agreement between the reference heart rate values (computed from the ECG and PPG) and the camera estimates during night-time hours, comprising a total estimated time of approximately 60.4 hours.

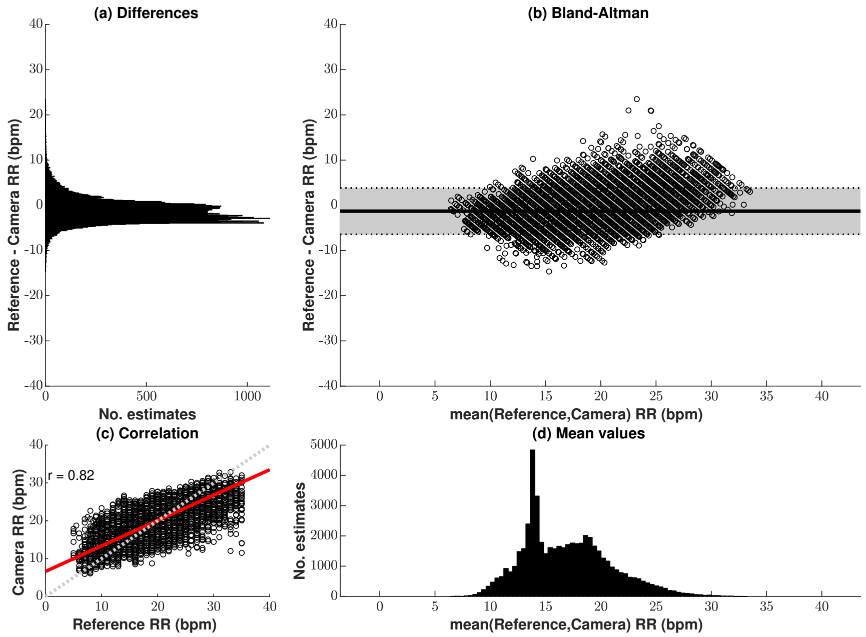


**Supplementary Figure 5.** Agreement between the reference respiratory rate values (computed from the IP) and the camera estimates during night-time hours, comprising a total estimated time of approximately 74.7 hours.

## References


1. Alonso, E. *et al.* Reliability and accuracy of the thoracic impedance signal for measuring cardiopulmonary resuscitation quality metrics. *Resuscitation* **88**, 28–34 (2015).

2. for the Advancement of Medical Instrumentation, A. *et al.* Cardiac monitors, heart rate meters, and alarms. *Am. Natl. Standard (ANSI/AAMI EC13: 2002) Arlington, VA* 1–87 (2002).

3. Jorge, J. *et al.* Non-contact assessment of peripheral artery haemodynamics using video infrared thermography. *IEEE Transactions on Biomed. Eng.* 1–1 (2020).
